# The lymphocyte function of allogeneic peripheral blood stem cell grafts is associated with relapse and cytomegalovirus reactivation

**DOI:** 10.1111/trf.70374

**Published:** 2026-08-20

**Authors:** Anna Söderström, Tengyu Wang, Johan Törlén, Hannes Lindahl, Davide Valentini, Stephan Mielke, Klara Asplund Högelin, Rebecca Axelsson Robertson, Michael Uhlin

**Affiliations:** ^1^ Department of Clinical Immunology and Transfusion Medicine Karolinska University Hospital Stockholm Sweden; ^2^ Department of Medicine Huddinge Karolinska Institutet Stockholm Sweden; ^3^ Cell Therapy and Allogeneic Stem Cell Transplantation, Karolinska Comprehensive Cancer Center Karolinska University Hospital Stockholm Sweden; ^4^ Department of Laboratory Medicine Karolinska Institutet Stockholm Sweden

**Keywords:** allogeneic hematopoietic stem cell transplantation, immune competence, lymphocyte proliferation assay, peripheral blood stem cell graft

## Abstract

**Background:**

Allogeneic hematopoietic stem cell transplantation (HSCT) is a treatment primarily for hematological malignancies. Infections, relapse, and graft versus host disease (GvHD) are some of the most common adverse events following HSCT. We aimed to evaluate whether the lymphocyte function of the stem cell graft was associated with these events.

**Study Design and Methods:**

We included peripheral blood stem cell (PBSC) grafts from 53 donors to HSCT recipients. The grafts' lymphocyte function was assessed by in vitro blast formation, expression of activation markers, and cytokine production upon stimulation. The cellular composition of the grafts was determined with flow cytometry. Results were compared with the incidence of infections, GvHD, relapse, and survival in the HSCT recipients.

**Results:**

We found an association between lower graft T cell response upon mitogen or superantigen stimulation and cytomegalovirus reactivation (*p* = .013 and *p* = .0028). Recipients of grafts with an above‐median programmed death‐1 (PD‐1) expression on CD8^+^ T cell blasts had a higher incidence of relapse and lower overall survival (*p* = .02). Both findings remained significant in the multivariable analysis.

**Discussion:**

Measurement of the lymphocyte response upon mitogen stimulation of PBSC grafts might provide beneficial knowledge of graft‐related factors that contribute to the incidence of complications after HSCT.

**Abbreviations**
7‐AAD: 7‐aminoactinomycin D
AA: aplastic anemia
ACD‐A: acid citrate dextrose solution A
ADV: adenovirus
AF750: alexa fluor 750
aGvHD: acute graft‐versus‐host disease
ALL: acute lymphoblastic leukemia
AML: acute myeloid leukemia
APC: allophycocyanin
ATG: anti‐thymocyte globulin
BV421/BV605/BV786: brilliant violet 421/605/786
CD: cluster of differentiation
cGvHD: chronic graft‐versus‐host disease
CML: chronic myeloid leukemia
CMML: chronic myelomonocytic leukemia
CMV: cytomegalovirus
CO_2_
: carbon dioxide
Cs: cyclosporine
Cy7: cyanine 7
EBMT: European Society for Blood and Marrow Transplantation
EBV: Epstein–Barr Virus
ELN: European LeukemiaNet
FACS: fluorescence‐activated cell sorting
FASCIA: flow cytometric assay of specific cell‐mediated immune response in activated whole blood
FITC: fluorescein isothiocyanate
G‐CSF: granulocyte colony‐stimulating factor
GvL: graft‐versus‐leukemia
HLA: human leukocyte antigen
HSA: human serum albumin
HSCT: hematopoietic stem cell transplantation
HSV: herpes simplex virus
IFN: interferon
IL: interleukin
IPSS: International Prognostic Scoring System
ISHAGE: International Society of Hematotherapy and Graft Engineering
MAC: myeloablative conditioning
MDS: myelodysplastic syndrome
MF: myelofibrosis
MIPSS70: Mutation‐Enhanced International Prognostic Scoring System
MM: multiple myeloma
MMF: mycophenolate mofetil
Mtx: methotrexate
NK: natural killer
OR: odds ratio
PBS: phosphate‐buffered saline
PBSC: peripheral blood stem cell
PC7: phycoerythrin–cyanine 7
PD‐1: programmed cell death protein 1
PD‐L1: programmed death‐ligand 1
PE: phycoerythrin
PerCP‐Cy5.5: peridinin chlorophyll protein–cyanine 5.5
PWM: pokeweed mitogen
RIC: reduced‐intensity conditioning
RT: room temperature
SCID: severe combined immunodeficiency
SEA: staphylococcal enterotoxin
Siro: sirolimus
TBI: total body irradiation
TCR: T‐cell receptor
TNF‐α: tumor necrosis factor alpha
URD: unrelated donor
Zol: zoledronate

## 1 BACKGROUND

Allogeneic hematopoietic stem cell transplantation (HSCT) is a potentially curative treatment for hematological diseases. The HSCT and the subsequent period of immunodeficiency entail a risk of severe adverse events, including infections, relapse, and graft‐versus‐host disease (GvHD).^1^ The stem cell graft is known to play an essential role in the complications mentioned above. However, there are currently limited assays for characterizing the stem cell graft, aside from cell dose and viability. We aim to explore whether lymphocyte proliferation following mitogen stimulation can be used to understand graft function.

Peripheral blood stem cells (PBSCs) are the most common graft source in HSCT.^2^ In addition to CD34^+^ hematopoietic stem cells, the graft contains numerous other cells of the hematopoietic system and lineages. Essential for the graft‐versus‐leukemia (GvL) effect are the mature graft T cells, which exceed the stem‐cell dose in the graft by multiple times.^3^, ^4^ These cells dominate the T cell compartment in the first months after transplantation and are responsible for both the GvL effect and protection against pathogens, as well as for acute GvHD.^5^ The content of T cells in the graft has been correlated with both the incidence of severe GvHD and superior overall survival, demonstrating the relationship between GvHD and GvL.^6^

The GvL effect is highly warranted given the high mortality rate associated with relapse after HSCT.^7^ Several previous studies have sought to identify biomarkers in patients and grafts to predict relapse risk and to identify targets to enhance the GvL effect without severe GvHD.^8^ Immune checkpoint signaling has been shown to play a role in many malignancies, in which tumors evade immune activation and subsequent killing of tumor cells.^9^ Programmed cell death 1 (PD‐1) and its ligand PD‐L1 are known to contribute to immune escape in many hematological malignancies and subsequent relapse.^10^

Beyond the number of cell types in a graft, graft fitness measured by viability is the only functional feature known to be important for successful transplantation.^11^ The function of mature graft cells is otherwise used in the adoptive transfer of virus‐specific T cells post‐HSCT to combat serious viral infections.^12^ Even though the immune function of the transplanted cells is central to the success of the treatment, a thorough assessment of the graft's general and specific immune competence and its relation to transplant‐related outcomes is poorly studied.

As a novel approach in this study, we aimed to evaluate whether graft fitness, defined as ex vivo T lymphocyte activation and function in response to specific and general stimuli, is associated with infections, GvHD, relapse, and survival in HSCT recipients.

## 2 STUDY DESIGN AND METHODS

### 2.1 Study population

We included PBSC grafts from 53 donors for recipients who underwent HSCT at Karolinska University Hospital in Stockholm, Sweden, between 2020 and 2022. Patients were enrolled randomly, and the cohort is representative of diagnoses and age within the HSCT population at our center and internationally. The study was conducted in accordance with the Declaration of Helsinki and approved by the regional ethics review board in Stockholm (date: 2020‐08‐26 and 2024‐11‐18, reference number: 2020‐01814 and 2024‐07607). Informed consent was obtained from all patients.

### 2.2 HSCT procedures

The conditioning regimen was administered according to standard protocols at the transplant center, based on diagnosis, patient age, comorbidities, and the human leukocyte antigen (HLA) match (Table 1). All donors were matched unrelated (URD), and all patients received anti‐thymocyte globulin (ATG) intended for in vivo T cell depletion in addition to the conditioning regimen to decrease the risk of rejection and GvHD.^13^, ^14^

**TABLE 1:** Patient characteristics.

Characteristics	*N* = 53 (%)
Donor sex	
Female	15 (28)
Male	38 (72)
Donor age	Mean: 29, range 19–54
Patient age	Mean: 58, range 0–74
Patient sex	
Female	19 (36)
Male	34 (64)
Diagnosis	
AA	1
ALL	3
AML	14
Lymphoma	4
CML	2
CMML	1
MDS	22
MF	2
MM	1
Myelosarcoma	2
SCID	1
CMV match	
R+/D+	27
R+/D−	11
R−/D+	2
R−/D−	13
Conditioning	
MAC	9
RIC	41
Other	3
GvHD prophylaxis	
Cs/Mtx	49
Grafalon/MMF/Cs	1
MMF/Siro	1
Mtx	1
Siro	1
TBI	
No	48
Yes	5

Abbreviations: AA, aplastic anemia; ALL, acute lymphoblastic leukemia; AML, acute myeloid leukemia; CML, chronic myeloid leukemia; CMML, Chronic myelomonocytic leukemia; CMV, cytomegalovirus; Cs, cyclosporin; GvHD, graft versus host disease; MAC, myeloablative conditioning; RIC, reduced‐intensity conditioning; MDS, myelodysplastic syndrome; MF, Myelofibrosis; MM, Multiple myeloma; MMF, mycophenylate mofetil; Mtx, methotrexate; SCID, severe combined immunodeficiency; Siro, sirolimus; MM; TBI, total body irradiation.

PBSCs were collected from URD after granulocyte‐colony stimulating factor (G‐CSF) mobilization via apheresis using the anticoagulant Citrate dextrose (ACD‐A). After collection, grafts were stored at 2–6°C and analyzed within 48 h of collection.

GvHD prophylaxis consisted of cyclosporine plus methotrexate for the majority of recipients. Acute GvHD (aGvHD) was graded from grade 0 (not present) to IV, according to the previously mentioned criteria.^15^ Acute GvHD Grade II to IV was considered severe aGvHD. Chronic GvHD (cGvHD) was graded according to the National Institute of Health criteria for clinical trials.^16^

### 2.3 Infections

All infectious complications occurring within the first 3 months after transplantation were documented. Cytomegalovirus (CMV)/Epstein Barr virus (EBV) reactivation was defined as detectable viral DNA (>500 IU/mL) in the blood on consecutive days in pre‐transplant seropositive recipients or in seronegative recipients with a seropositive donor. Viral, fungal, and bacterial diseases were defined as the presence of detectable pathogens in blood or tissue specimens, together with clinical symptoms.

### 2.4 Cellular composition

Leukocyte count was assessed using an automated blood cell counter (Sysmex XP300, Kobe, Japan). CD34^+^ cell enumeration was performed using a single‐platform analysis based on the ISHAGE gating strategy.^17^ Briefly, 1 million nucleated cells were added to duplicate tubes with Trucount beads (BD, Stockholm, Sweden) and incubated for 10 min at room temperature (RT) with 7AAD (Immunotech A07704), CD34‐PE (HPCA2:8G12 [IgG1], BD), and CD45‐FITC (2D1 [IgG1], BD). For lymphocyte phenotyping, CD3‐FITC (SK7), CD16‐PE (B73.1), CD56‐PE (NCAM16.2), CD45‐PerCP‐Cy™5.5 (2D1), CD4‐PE‐Cy™7 (SK3), CD19‐APC (SJ25C1), CD8‐APC‐Cy7 (SK1), CD25‐BV605 (2A3) and CD127‐BV421 (HIL‐7R‐M21) were used (BD). After that, 1 mL of lysing solution (IOTest3 Lysing Solution; diluted 1:10 with deionized water) was added and incubated for an additional 10 min at room temperature. Cells were immediately analyzed by flow cytometry (DxFlex, Beckman Coulter, Brea, CA, USA). The results were analyzed in the Kaluza software (Beckman Coulter).

### 2.5 Lymphocyte proliferation assay

We used a modified version of the Flow cytometric Assay of Specific Cell‐mediated Immune response in Activated whole blood (FASCIA) to evaluate graft lymphocyte proliferation.^18^ Briefly, 10 × 10^6^ leukocytes were obtained from the graft and washed in phosphate‐buffered saline (PBS) to remove the ACD‐A used during graft collection. Ten milliliters of 5% human albumin was added to the cells and allowed to rest for 5 min before centrifugation and resuspension in a medium consisting of RPMI 1640, penicillin, streptomycin, L‐glutamine, AB‐serum, and calcium. The cells in the medium were stimulated in 9 separate sterile polypropylene Falcon 12 × 75 mm FACS tubes with cap (BD Biosciences, CA, USA) with the general stimuli pokeweed mitogen (PWM), and the superantigen staphylococcal enterotoxin A and B (SEA/SEB) (Sigma‐Aldrich, St. Louis, MO, USA), the specific pathogen antigens Candida Albicans (Greer Laboratories, Lenoir, NC, USA), CMV, EBV, Adenovirus (ADV), Herpes simplex virus type 1 (HSV‐1) (Microbix Biosystems Inc., Toronto, Canada), Zolendronate (Zol) (Medac GmbH, Wedel, Germany) + Interleukin‐2 (IL‐2) or medium. The cells were incubated for 7 days at 37°C, 5% CO_2_, and 95% humidity. The supernatant was separated and stored at −70°C until cytokine analysis. The stimulated cells were stained with PD‐1‐PE (EH12.1), HLA‐DR‐BV604 (G46‐6), CD3‐BV786 (SK7) (BD Biosciences, San Jose, CA, USA), CD19‐PC7 (J3‐119), CD4‐APC‐AF750 (13B8.2) (Beckman Coulter), TCR‐g/d‐APC (11F2) (Miltenyi Biotec, Bergisch Gladbach, Germany). Samples were incubated for 10 min, followed by lysis of erythrocytes, wash, and resuspension in 500 μL PBS‐HSA. The concentration of blasts defined by increased forward scatter compared to resting cells was acquired with a DxFlex flow cytometer (Beckman Coulter). Samples were analyzed in the Kaluza software (Beckman Coulter). The gating strategy is presented in Figure S1.

### 2.6 Cytokine analysis

The concentration of cytokines was evaluated in the supernatant from the lymphocyte proliferation assay at day seven, following stimulation with PWM, Candida Albicans, CMV, EBV, HSV, and Zol + IL‐2, as well as in the medium. The analyzed cytokines were Interleukin (IL)‐1β, IL‐2, IL‐6, IL‐10, IL‐17a, tumor necrosis factor (TNF) α, and interferon (IFN) γ. The analysis was performed using a MilliPlex MAP Human Cytokine/Chemokine/Growth Factor Panel A Multiplex assay (HCYTA‐60 K‐07C, Merck KgAA, Darmstadt, Germany) and analyzed on a Luminex 200 with xPONENT 4.3 (Luminex Corporation) using the manufacturer's protocol. The data were analyzed in the Belysa software package v1.1.0 (Merck Millipore). Two normalized cytokine analyses were used. Adjusted cytokine activity per blast or T cell was calculated by dividing the cytokine activity by the number of blasts or T cells for the specific tube. Cytokine response, measured as a percentage of the maximum PWM response, was correlated with both cell proliferation and clinical outcome.

### 2.7 Statistical analysis

The Mann–Whitney *U* test was used to compare lymphocyte counts and recipient/donor characteristics. Spearman correlation was used for nonparametric correlations. Categorical parameters were compared using the *χ* ^2^ test, unless there were fewer than 5 cases in the groups, in which case Fisher's exact test was used. Survival was estimated using the Kaplan–Meier method and compared using the log‐rank test. Between‐group differences in relapse and GvHD were illustrated using cause‐specific cumulative incidence curves and quantified using cause‐specific Cox regression models, with censoring for competing events.

Cause‐specific Cox regression was used for multivariable analysis of relapse.^19^ A series of models adjusting for clinically relevant factors known to influence the outcome, such as donor and recipient age, conditioning regimens, HLA matching, acute and chronic GvHD, and disease risk, were included in the analysis. Disease risk was defined as high or intermediate based on standard criteria for each hematological disease, for example, validated risk models such as ELN 2022, IPSS, MIPSS70, cytogenetics, and hematological remission status at the time of transplantation. Factors with *p*‐values <.50 in the univariable analysis were subsequently included in the backward‐elimination multivariable analysis.

CMV reactivation was analyzed using logistic regression, with reactivation within 3 months post‐HSCT as the primary outcome. Variables with *p* < .5 in the univariable analysis were included in the multivariable analysis. These variables included T cell blast count after SEA stimulation, donor/recipient CMV status, and disease severity (see Table 3). A similar strategy was used for a series of sensitivity analyses of clinically relevant confounders (Table S1).

A *p*‐value cutoff of <.05 was considered significant for all analyses. Statistical analysis was performed with R version 4.4.1 (R Core Team 2024, Vienna, Austria).

## 3 RESULTS

Patients' characteristics are found in Table 1. In brief, 51/53 (96%) of patients were adults with malignant disease. All donors were URD, and all patients received ATG in addition to their conditioning regimen. The median follow‐up time was 825 days (72–1360 days). The incidence of post‐transplant complications is shown in Table 2

**TABLE 2:** Post‐transplant complications.

Outcome	*N* (%)
Relapse	
No	35 (66)
Yes	18 (34)
aGvHD	
Grade 0–I	34 (64)
Grade II–IV	19 (36)
cGvHD	
No	40 (75)
Yes	13 (25)
Survival status	
Alive	40 (75)
Deceased	13 (25)

Abbreviations: aGvHD, acute graft‐versus‐host disease; cGvHD, chronic graft‐versus‐host disease.

### 3.1 Comparison between serostatus and lymphocyte response

For robustness testing of our lymphocyte proliferation assay, the serostatus for CMV and EBV was compared with the lymphocyte response upon stimulation with the corresponding antigen. The concentration of T cell blasts upon CMV stimulation was significantly higher (*p* < .05) in CMV‐positive donors. While all CMV‐negative donors showed a low response, some seropositive donors also responded poorly to CMV stimulation. The IFNγ response after CMV stimulation was likewise higher in CMV‐positive donors compared to CMV‐negative donors (Figure S2). CMV reactivation in recipients of seropositive donors occurred in 30% of cases. The incidence of CMV reactivation among recipients of donors with low or high T cell blast formation to the antigen was similar (5 vs. 2 patients, *p* = .4). For EBV, only six donors were EBV‐negative, and no significant difference in mean T cell blast formation was found between seropositive and seronegative donors.

### 3.2 Clinical outcomes

#### 3.2.1 Infections

Patients with reactivated CMV had grafts with a significantly lower response in the lymphocyte proliferation assay to PWM and SEA stimulation than those without reactivation. This was evident in the parameters of T cell blast formation and in the expression of the activation marker HLA‐DR on T cells (Figures 1 and 2). After multivariable adjustment, low T cell blast formation upon SEA stimulation remained an independent risk factor for CMV reactivation (Table 3, Table S1).

**FIGURE 1:**
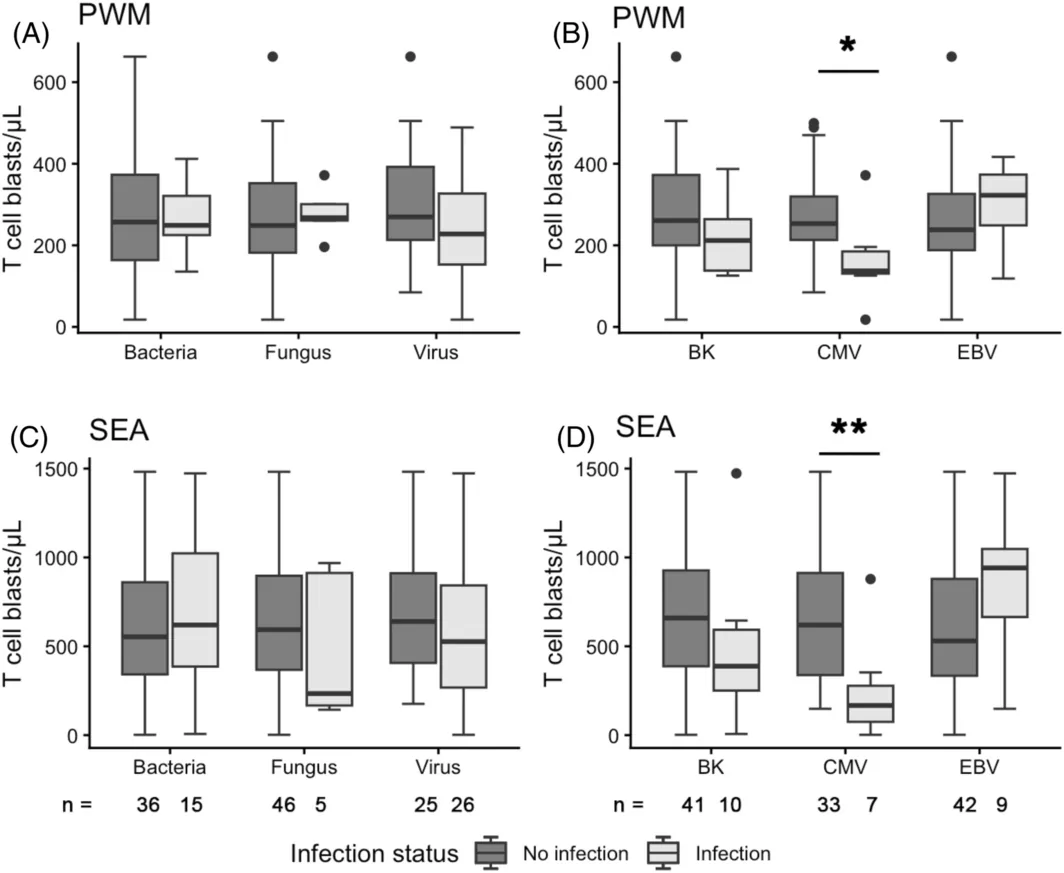
CD3^+^ blast formation after pokeweed mitogen (PWM) and Staphylococcal enterotoxin A (SEA) stimulation. (a and c) Differences in the concentration of T cell blasts after stimulation with SEA/PWM between patients with or without bacterial, fungal, or viral infections/reactivations within 3 months post‐transplantation. (b and d) Differences in the concentration of T cell blasts after stimulation with SEA/PWM between patients with or without the most common viral reactivations within 3 months post‐transplantation (human polyomavirus 1 BK, cytomegalovirus [CMV], Epstein Barr virus [EBV]). T cell blast concentration after PWM and SEA stimulation correlated with CMV reactivation within 3 months post‐transplantation. *p < .05, **p < .01.

**FIGURE 2:**
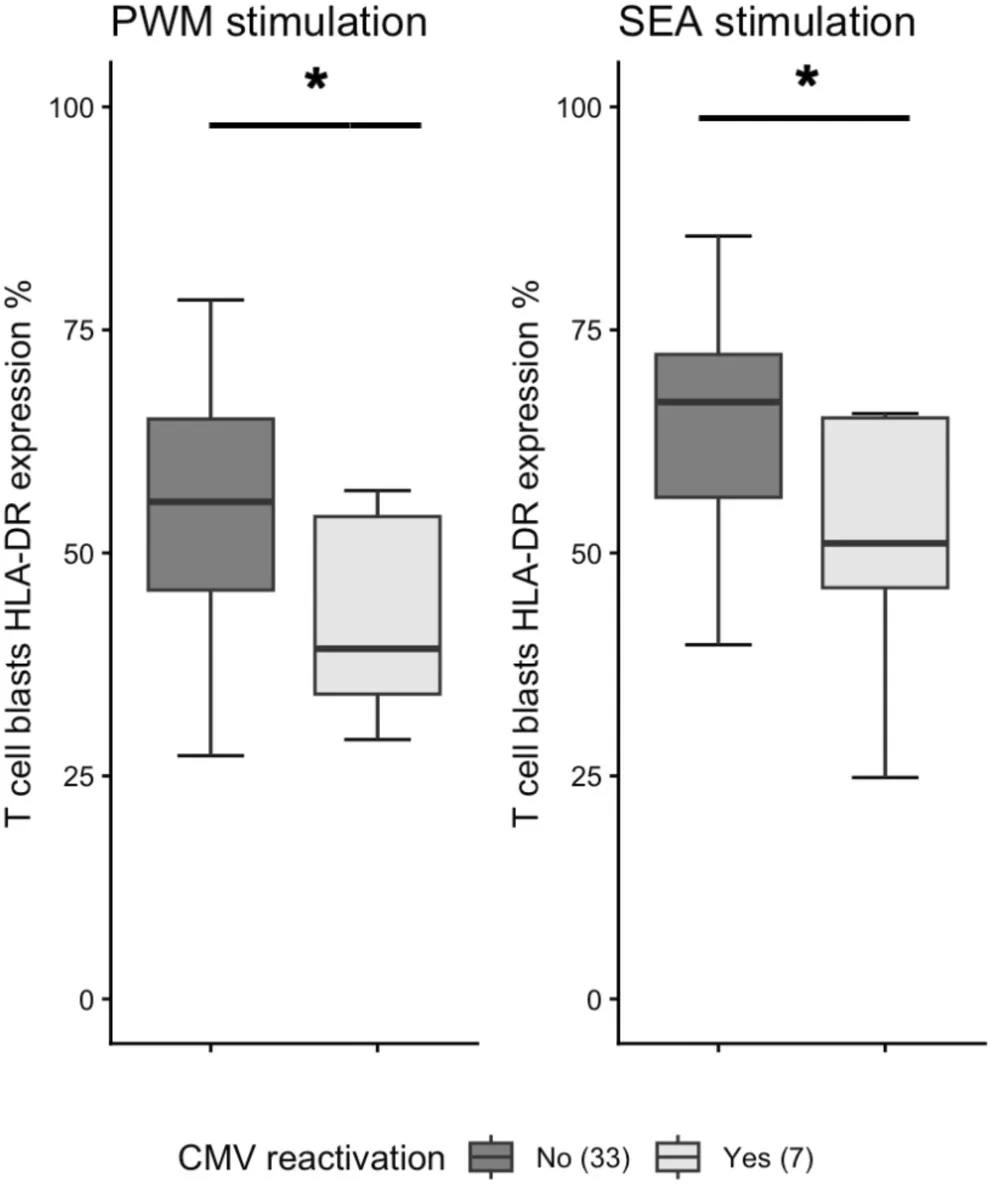
Human leukocyte antigen (HLA)‐DR expression on T cells (T cell activation) after pokeweed mitogen (PWM) and staphylococcal enterotoxin A (SEA) stimulation. Lower HLA‐DR expression on T cells in the graft after PWM (*p* = .03) and SEA (*p* = .04) stimulation correlated with a higher incidence of cytomegalovirus (CMV) reactivation. *p < .05.

Stimulation with the specific γδ T cell stimulus Zolendronate + IL‐2 in the lymphocyte response assay also resulted in a lower concentration of γδ T cells in grafts to CMV reactivating recipients (150 vs. 20 cells/μL, *p* = .05) (Figure S3). Of note, the blast formation, cytokine release, and expression of activation markers upon CMV stimulation were not associated with the reactivation of CMV.

The most common pathogens detected in our cohort were reactivation of CMV (*n* = 7), EBV (*n* = 9), and human polyomavirus 1 (BK virus) (*n* = 10), with or without viral disease. No other infections were correlated with the lymphocyte response (blast formation, cytokine release, or upregulation of activation markers), whether with specific stimuli or with SEA/PWM.

#### 3.2.2 Survival and relapse

Relapse rates were significantly higher in patients with grafts exhibiting higher PD‐1 expression on CD8^+^ T cells after PWM stimulation (18% vs. 50%, *p* = .02). Survival was also significantly lower in patients with grafts showing high PD‐1 expression after PWM stimulation (Figure 3). Relapse was by far the most common cause of death (85%), and only 2 cases of transplant‐related mortality occurred in this cohort. Graft PD‐1 expression did not correlate with the risk of acute or chronic GvHD. In the multivariable analysis, graft PD‐1 expression after PWM stimulation remained the strongest predictor of relapse (*p* = .02). In addition to PD‐1 expression, donor age and disease risk were strong predictors of relapse (Table 3). In a series of sensitivity analyses, PD‐1 remained a strong factor across different models, with disease severity, donor age, and other clinically relevant confounders for relapse included (Table S1).

**FIGURE 3:**
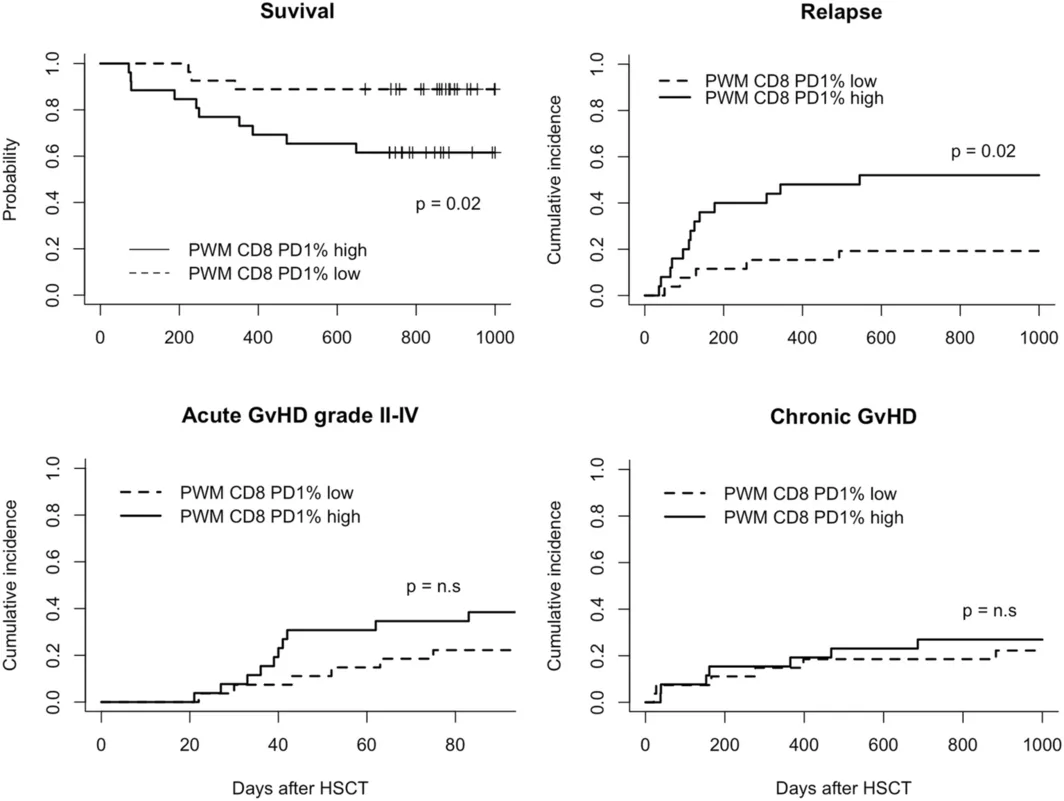
Clinical outcome and CD8^+^ T cell programmed death‐1 (PD‐1) expression upon pokeweed mitogen (PWM) stimulation. A significantly higher risk of relapse and lower overall survival were observed in patients whose grafts showed high PD‐1 expression on CD8^+^ T cells upon PWM stimulation in the lymphocyte proliferation assay (*p* = .02). There was no difference in the development of graft versus host disease (GvHD). The relapse graph shows patients with malignant disease only (*n* = 51). HSCT, hematopoietic stem cell transplantation. n.s, not significant.

**TABLE 3:** Multivariable analysis with backward elimination.

(a) Cause‐specific Cox regression				
Relapse	Univariable model, hazard‐ratio (95% CI)	*p* Value	Multivariable model, hazard‐ratio (95% CI)	*p* Value
PD‐1 CD8 (%)	1.05 (1.02–1.08)	.003*	1.04 (1.02–1.07)	.02*
Donor age	0.93 (0.88–0.99)	.03*	0.95 (0.89–1.01)	.1
HLA matching	0.70 (0.32–1.55)	.38		
Acute GvHD	0.81 (0.47–1.43)	.48		
Chronic GvHD	0.50 (0.15–1.73)	.28	0.43 (0.12–1.51)	.19
Disease severity	3.23 (0.74–14.1)	.12		

(b) CMV reactivation logistic regression model				
CMV reactivation	Univariable model, OR (95% CI)	*p* Value	Multivariable model, adjusted OR (95% CI)	*p* Value
SEA CD3 blasts (/100 units)	0.62 (0.36–0.87)	.02*	0.59 (0.36–0. 99)	.045*
CMV status donor pos	2.61 (0.37–52.6)	.40		
CMV status recipient pos	0.18 (0.01–5.17)	.26		
Disease severity	0.09 (0.01–0.52)	.01*	0.06 (0.01–0.55)	.014*

*Note*: Disease severity (intermediate/high risk). Alternative models for sensitivity analysis are provided in Table S1.

Abbreviations: CI, confidence interval; CMV, cytomegalovirus; GvHD, graft‐versus‐host disease; HLA, human leukocyte antigen; OR, odds ratio; PD‐1, programmed cell death protein 1; SEA, staphylococcal enterotoxin A.

* *p* < .05.

### 3.3 Cellular graft composition

The mean dose of CD34^+^ stem cells was 7.2 × 10^6^/kg (2.9–10). The administered CD34^+^ cell dose was capped at 10 × 10^6^ CD34^+^ stem cells/kg per the center's standard operating procedures; higher harvested doses were reduced upon arrival. Doses of other cell types also varied widely between grafts (Table 4). The starting T cell dose in the graft did not correlate with lymphocyte proliferation in response to PWM, SEA, or specific antigens. We found no associations between graft cell doses and the incidence of infections, GvHD, relapse, or survival (data not shown).

**TABLE 4:** Cell doses in grafts.

Cell dose × 10^6^/kg	Mean	Range
CD34^+^	7.2	2.9–10.0
T cells	273.2	132–653
CD4^+^ T cells	165	55–371
B cells	70.2	24–229
NK cells	32.3	8–73
Platelets	6283	2120–17,329

### 3.4 Cytokine response

The cytokine response of the lymphocyte proliferation assay is illustrated with overall survival in Figure S4. Single cytokine responses did not correlate with lymphocyte proliferation measured in blast formation, T cell concentration in the starting material, or T cell activation markers (data not shown). Absolute cytokine production and cytokine production per T cell blast did not correlate with any clinical outcome. The production of cytokines upon specific antigen stimulations expressed as a percentage of PWM‐stimulated cytokine levels did not correlate with infectious complications (data not shown).

## 4 DISCUSSION

HSCT still entails a considerable risk for adverse events such as relapse and infections, and factors influencing the incidence are being studied to improve prediction and prevention.^20^, ^21^, ^22^ While most prior studies focus on patient‐associated biomarkers or graft cell composition, we investigated the fitness of the PBSC graft and its relationship to post‐HSCT outcomes. We tested the graft lymphocyte response to PWM, SEA, and specific pathogen‐derived antigens. Our main findings were: (1) Inferior graft T cell proliferation and activation upon PWM and SEA stimulation were associated with CMV reactivation, and (2) Recipients of grafts with higher expression of PD‐1 on CD8^+^ T cells upon PWM stimulation had a higher incidence of relapse and lower overall survival.

Infectious complications are common after HSCT despite various precautions. We investigated the incidence of infections during the first 3 months after HSCT, where the expanded mature graft T cells dominate the patients' T cell population.^23^ CMV infections still contribute to significant morbidity in HSCT recipients, even after the introduction of the preemptive treatment Letermovir.^24^ We observed that an inferior graft T cell response upon PWM and SEA stimulation was associated with CMV reactivation. The lymphocyte response to PWM and other nonspecific stimuli is low in different immune‐compromised settings.^18^ A robust PWM or SEA response depicts a vast population of functional lymphocytes ready to proliferate and perform effector functions. Our data suggest that responses to nonspecific stimuli, such as PWM and SEA, could serve as fitness markers for the graft. Although the potential mechanistic relationship is not investigated in our study, it warrants further exploration in future studies. Interestingly, the graft T cell response upon CMV stimulation was not associated with CMV reactivations. The mechanisms by which these stimuli activate lymphocytes differ significantly. While PWM and SEA bypass the regular activation pathway and stimulate T cells regardless of specificity, depicting global T cell function, specific antigens need to be presented on HLA by antigen‐presenting cells to virus‐specific T cells. The CMV‐specific antigens used contained a peptide mix spanning early to late viral replication, which should stimulate a broad array of CMV‐reactive T cells. Due to the nature of the antigen, virus‐specific peptides would, to a greater extent, activate αβ T cells rather than γδ T cells and NK cells, resulting in a lack of readout for several important components of the graft for early viral control. In addition, the lymphocyte proliferation assay might benefit from optimization for CMV‐specific stimulation; improvements could include optimizing stimulation and peptide composition, and determining cutoffs to improve sensitivity and specificity. Other factors known to influence the risk for CMV, such as GvHD and its treatment, and the use of ATG might also influence the incidence of reactivation.^25^ We also found reduced γδ T cell proliferation in the grafts of CMV‐reactivating patients. This finding is consistent with other studies showing that γδ T cells can recognize CMV and contribute to the immune response against the virus.^26^, ^27^

Understanding the risk of relapse is essential when transplanting patients with malignant diseases. Several factors are known to influence the risk of relapse, including disease burden and intensity of the conditioning regimen.^28^ Donor/graft‐related risk factors are less well studied. Our data shows that higher PD‐1 expression on graft CD8^+^ T cells after PWM stimulation correlated with a higher risk of relapse in the recipient. PD‐1 is upregulated upon T cell activation but also functions as an inhibitor of TCR signaling. A high PD‐1 expression is observed in exhausted T cells, although PD‐1 expression alone does not define T cell exhaustion.^29^, ^30^, ^31^ To our knowledge, this is the first study to evaluate PD‐1 expression in donor grafts after PWM/SEA stimulation in relation to relapse. Previous publications have evaluated PD‐1/PD‐L1 expression and interaction in both ex vivo models and post‐transplant settings.^9^, ^32^, ^33^, ^34^ These studies conclude that a high expression of PD‐1 or other exhaustion markers post‐HSCT was associated with relapse and death. The higher PD‐1 expression we found to correlate with increased relapse incidence in patients may reflect differences in T cell differentiation status or immune regulation, but the underlying mechanisms and potential relationship with relapse remain unclear. Additionally, the in vivo PD‐1 expression post‐HSCT is also influenced by multiple factors including lymphopenia, T cell depletion, disease status and inflammation and likely not solely graft‐intrinsic.^35^ Studies comparing graft PD‐1 expression and additional activation markers with in vivo post‐HSCT measurements are needed to further clarify potential associations.

We could not find an association between the dose of any cell type in the graft and the studied clinical outcomes. Several earlier studies have found an increased incidence of aGvHD with higher T cell doses, but we could not confirm this in our cohort.^36^, ^37^ A reasonable explanation is that, as a standard at our center, recipients of unrelated donors receive ATG in addition to their conditioning regimen as GvHD prophylaxis. In addition, only one recipient in our cohort experienced grade III‐IV aGvHD. ATG can remain in the bloodstream for weeks to months after transplantation and might also hamper the positive effects of a larger dose of mature graft T cells, such as protection against infections, which was likewise not found in our study.^38^

We recognize that this is a hypothesis‐generating, single‐center study, and larger studies are warranted to validate our findings. Our main findings remained significant in the multivariable analysis. However, it should still be stressed that the cohort is relatively small and diverse. Both type I and especially type II errors are possible in analyses with few events, such as CMV reactivation. A comparison of lymphocyte function in an ex vivo setting pre‐HSCT, combined with post‐HSCT measurements of proliferative responses, PD‐1 expression dynamics, and immune recovery, would be valuable in future studies to strengthen the mechanistic interpretation. Nevertheless, we believe that a better understanding of differences in graft function could improve the understanding of mechanisms contributing to post‐transplantation complications. Developing pre‐HSCT assays for potential risk‐stratification could be useful.

In conclusion, we evaluated whether PBSC graft fitness, as measured by an adapted lymphocyte proliferation assay, was associated with outcomes after HSCT. CMV reactivation and relapse, as well as overall survival, were associated with the grade of T cell proliferation and activation upon stimulation. Evaluation of graft fitness in addition to graft cell content might provide beneficial knowledge of donor‐related factors that play a role in the incidence of complications after HSCT. Assessment of graft fitness could be of interest for further exploration and refinement in larger studies.

## AUTHOR CONTRIBUTIONS

TW, AS and MU designed the study. AS and TW performed the laboratory analyses, data management, and wrote the manuscript. KAH and RAR contributed with specialist knowledge on the laboratory assays. DV and SM provided registry data and interpretation of this. HL and JT gave valuable feedback throughout the entire process. All authors contributed to drafting the article and approved the final version of the manuscript.

## FUNDING INFORMATION

This work was supported by the following grants: Swedish Childhood Cancer Foundation (PR2022‐0066), Swedish Cancer Society (2023‐01988), Swedish Research Council (2021‐01755), Cancer Research Foundation of Radiumhemmet (Uhlin), and Stockholm County Council (2022‐PR00132).

## CONFLICT OF INTEREST STATEMENT

The authors have disclosed no conflicts of interest.

## Supporting information


**Figure S1.** Gating strategy for the lymphocyte proliferation assay.
**Figure S2.** (a) IFNγ production upon CMV stimulation based on donor CMV status. (b) IFNγ adjusted per T cell based on donor CMV status. ***p < .001 (donor negative *n* = 29, postive *n* = 24).
**Figure S3.** γδ T cell proliferation was significantly higher in donor grafts to recipients who reactivated CMV. *p < .05 (CMV reactivation Yes *n* = 7, No *n* = 33).
**Figure S4.** Heatmap of cytokine production after mitogen and antigen stimulation. The three panels show cytokine concentrations after stimulation with PWM, CMV, and Candida. Each row represents a study subject, with the z‐score of the cytokine concentration. The left column in each panel shows patient survival. No clustering of cytokine production was observed in relation to survival.
**Table S1.** Cause‐specific Cox regression models–sensitivity analysis.

## Data Availability

The data that support the findings of this study are available from the corresponding author upon reasonable request.
