# Supplementary material for: The lymphocyte function of allogeneic peripheral blood stem cell grafts is associated with relapse and cytomegalovirus reactivation
Source: Transfusion. 2026 Aug 20;66(11):2302–12. doi: 10.1111/trf.70374 (PMC13753063; doi:10.1111/trf.70374)
Supplement: Supplementary file 1 [file TRF-66-2302-s001.docx]

## Appendix

**Supplementary figure 1.** Gating strategy for the lymphocyte proliferation assay.

**Supplementary figure 2**

b)

a)

**Supp. 2.** a) IFNγ production upon CMV stimulation based on donor CMV status. b) IFNγ adjusted per T cell based on donor CMV status. *** p < 0.001 (Donor negative n = 29, postive n = 24)

**Supplementary figure 3.**

**Supp. 3.** γδ T cell proliferation was significantly higher in donor grafts to recipients who reactivated CMV. *; p <0.05 (CMV reactivation Yes n = 7 , No n = 33)

**Supplementary Figure 4.**

**Suppl. Fig 4.** Heatmap of cytokine production after mitogen and antigen stimulation. The three panels show cytokine concentrations after stimulation with PWM, CMV, and Candida. Each row represents a study subject, with the z-score of the cytokine concentration. The left column in each panel shows patient survival. No clustering of cytokine production was observed in relation to survival.

**Supplementary table 1.**

Cause specific Cox regression models – sensitivity analysis

| **Relapse** | Multivariable model  Proportional Hazard ratio (95% CI) | P-value |
| --- | --- | --- |
|  |  |  |
| **Model A**    T cell PD1% PWM  Donor age  HLA match | 1.04 (1.01 – 1.07)  0.96 (0.89 – 1.02)  0.77 (0.30 – 1.93) | .018 *  .19  .57 |
| **Model B** |  |  |
| T cell PD1% PWM  Donor age  Disease severity | 1.03 (1.00 – 1.06)  0.95 (0.89 – 1.01)  2.31 (0.50 – 10.75) | .05  .10  .28 |
| **Model C** |  |  |
| T cell PD1% PWM  Donor age  MAC    **Model D**    T cell PD1% PWM  Donor age  Chronic GvHD | 1.04 (1.01 – 1.07)  0.95 (0.89 – 1.02)  0.73 (0.21 – 2.52)        1.04 (1.01 – 1.07)  0.94 (0.89 – 1.01)  0.43 (0.12 – 1.51) | .015 *  .13  .61        .017 *  .10  .19 |

CMV reactivation logistic regression models – sensitivity analysis

| **CMV reactivation** | Multivariable model  Adjusted OR (95% CI) | P-value |
| --- | --- | --- |
|  |  |  |
| **Model A**    T cell blasts SEA  Donor CMV  Patient CMV | 0.57 (0.29 – 0.81)  1.93 (1.19 – 43.9)  0.05 (0.01 – 2.59) | .03 *  .60  .16 |
| **Model B** |  |  |
| T cell blasts SEA  Disease severity  HLA match    **Model C**    T cell blasts SEA  Disease severity  Acute GvHD | 0.60 (0.29 – 0.88)  0.05 (0.01 – 0.46)  0.88 (0.13 – 5.71)        0.59 (0.27 – 0.88)  0.05 (0.01 – 0.43)  0.60 (0.13 – 2.23) | .049 *  .014 *  .89        .057  .014  .47 |

**Suppl. Table 1: Clinically important confounders for relapse and CMV reactivation**. Abbreviations: PD-1, programmed cell death protein 1. MAC, myeloablative conditioning. GvHD, graft versus host disease. CMV – cytomegalovirus. SEA - staphylococcal enterotoxin A
